# Trait procrastination undermines outcome and efficacy expectancies for achieving health-related possible selves

**DOI:** 10.1007/s12144-019-00338-2

**Published:** 2019-06-27

**Authors:** Fuschia M. Sirois

**Affiliations:** 1 Department of Psychology, The University of Sheffield, 1 Vicar Lane, Sheffield, S1 2LT UK; 2 Department of Psychology, University of Windsor, Windsor, Canada

**Keywords:** Possible selves, Procrastination, Efficacy expectancies, Outcome expectancies, Health behaviour change

## Abstract

People often fail at following through with their health behaviour goals. How health goals are cognitively represented holds promise for understanding successful health behaviour change. Health-related possible selves (HPS) reflect cognitive representations of a future self that people may wish to achieve (hoped-for-HPS) or avoid (feared-HPS), that can promote health behaviour change. However, success depends on the strength of the efficacy and outcome expectancies for achieving/avoiding the HPS. Personality traits linked to poor self-regulation are often not considered when assessing the potential self-regulatory functions of HPS. The current study addressed this issue by examining the associations of trait procrastination with efficacy and outcome expectancies for hoped-for-HPS and feared-HPS, and health behaviour change intentions and motivations in a community sample (*N* = 191) intending to make healthy changes in the next 6 months. Trait procrastination was associated with weaker intentions and motivations for health behaviour change, and lower efficacy and outcome expectancies for hoped-for-HPS, but not feared-HPS. Bootstrapped multiple mediation analysis found significant indirect effects of procrastination on health behaviour intentions, through outcome, but not efficacy, expectancies for hoped-for-HPS. Results suggest that issues in imagining a hoped-for-HPS can be achieved are linked to weak intentions for health behaviour change for those with chronic self-regulation difficulties. Research into interventions that strengthen feeling connected to hoped-for-HPS is recommended.

## Introduction

Despite the best intentions, people often fail at following through with their health behaviour goals. For example, it is estimated that more than 50% of people stop using their gym memberships within 6 months of starting (CouponCabin.com 2012). Procrastinating on important health behaviour goals, such as increasing physical exercise and eating a healthier diet, can have a number of far-reaching and negative health consequences including increased risk for obesity, and the development of chronic health conditions including cardiovascular disease, cancer, diabetes and arthritis (World Health Organization 2015). Issues in self-regulation are well-known to contribute to failure with health behaviour goals (Hagger 2010), as are personality traits reflecting self-regulation difficulties (Sirois et al. 2003). However, the way in which individuals cognitively represent their health goals (Hooker and Kaus 1994), and their confidence in their ability to reach these goals (Bandura 1977; Hooker and Kaus 1994), are also important factors to consider for understanding successful health behaviour change.

Possible selves (Markus and Nurius 1986) are one type of cognitive representation that can be particularly beneficial for understanding whether or not individuals achieve their health behaviour goals. Possible selves theory (Markus and Nurius 1986) posits that individuals have a repertoire of different hoped-for and feared possible selves that reflect cognitive representations of current goals and provide incentives to motivate current behaviour. In this respect, possible selves can have an implicit self-regulatory function by highlighting discrepancies between the current and future possible selves, which in turn, can motivate approach or avoidance behaviours (Markus and Nurius 1986; vanDellen and Hoyle 2008). For example, a hoped-for possible self that is 10 pounds slimmer may motivate appropriate diet and exercise changes, and a feared possible self that has diabetes may motivate similar weight management behaviours. In this respect, possible selves within the health domain can provide meaningful incentives to direct health-relevant behaviour (Hooker 1992). An experimental study on the role of health-related possible selves (HPS) for promoting exercise behaviour supports this proposition. Participants who were asked to imagine a hoped-for-HPS or feared-HPS engaged in more exercise behaviour 4 and 8 weeks post-intervention compared to those in the control group (Murru and Martin Ginis 2010).

Yet envisioning a HPS may not be enough to achieve health goals if the self-regulatory processes associated with this HPS are weak. Hooker (1992) found that confidence that one could achieve or avoid HPS (efficacy expectancies), and perceptions of the likelihood that a HPS could be realised (outcome expectancies), were associated with better perceived health. Similarly, in a study of young and middle-aged adults, efficacy expectancies for attaining/avoiding hoped-for-HPS and feared-HPS was associated with the practice of health behaviours (Hooker and Kaus 1994). This evidence is consistent with Bandura’s self-efficacy theory (Bandura 1977), and suggests that, in the absence of strong efficacy and outcome expectancies, the health goals represented by HPS may not be achieved.

Chronic procrastination is one personality trait that has been linked to difficulties both in engaging in health-promoting behaviours, and envisioning a future possible self. Defined as a tendency to unnecessarily delay the start or completion of intended tasks despite awareness of the negative consequences of this delay (Ferrari and Tice 2000), procrastination when habitual, can be viewed as a trait-like quality characterised by chronic self-regulation difficulties and avoidance (Sirois 2016). Several studies have found that trait procrastination is associated with less frequent practice of, and weaker intentions to engage in, health-promoting behaviours such as physical activity and healthy eating (Argiropoulou et al. 2016; Sirois 2004, 2007, 2015; Sirois et al. 2003), and that less practice of such behaviours accounts in part for the poor physical health outcomes associated with this trait (Sirois 2007; Sirois et al. 2003). Theory and research also suggest that procrastination, whether momentary or chronic, reflects difficulty in envisioning the future (Sirois 2014), and that procrastinators feel disconnected to their future selves (Blouin-Hudon and Pychyl 2015, 2017; Sirois and Pychyl 2013).

Examining the hoped-for-HPS and feared-HPS of chronic procrastinators has the potential to provide important insights for understanding why people may procrastinate on their health behaviour goals. Such insights can contribute to the development of interventions to help bolster the motivations and intentions of people who chronically struggle to reach their health goals. Yet to date there is limited research into how HPS are linked to the self-regulatory processes involved in reaching health behaviour goals, and with respect to personality traits associated with self-regulation difficulties.

**Fig. 1:**
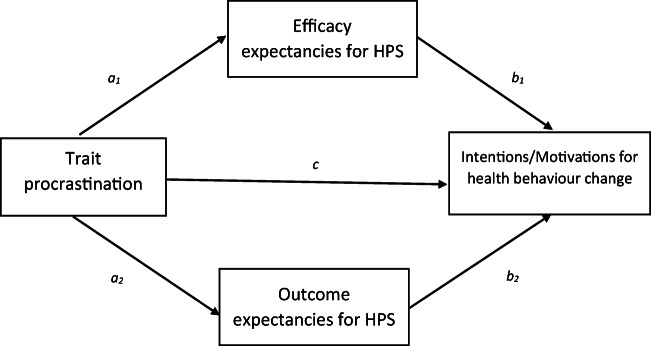
Proposed model of the indirect effects of trait procrastination on intentions and motivations for making health behaviour changes through efficacy and outcome expectancies for achieving hoped-for, and avoiding feared, health-related possible selves (HPS)

The aim of the current study was to investigate the associations of trait procrastination to the self-regulatory processes reflected in the efficacy and outcome expectancies for hoped-for-HPS and feared-HPS, and how these related to intentions and motivations among individuals intending to make health behaviour changes (see Fig. 1). Previous findings suggest differential self-regulatory processes for pursuing positive versus negative health goals, such that the steps needed to achieve positive health goals may be more salient than those for avoiding a negative health goal (Hooker 1992). Given this, and theory highlighting that difficulties in relating to a future self underscore procrastination (Sirois and Pychyl 2013), trait procrastination was expected to be associated with lower efficacy and outcome expectancies for achieving hoped-for-HPS, and avoiding feared-HPS, but that the association for feared-HPS would be weaker. In addition, it was expected that lower expectancies would explain the association between trait procrastination and weaker intentions and motivations to make health behaviour changes. Because previous research has found that trait procrastination scores are higher among lower age cohorts, and in males versus females (Beutel et al. 2016), both age and participant sex were added as covariates in the models tested.

## Methods

### Participants and Procedure

After obtaining ethical clearance for data collection from the University Research Ethics Board, 205 adults were recruited to participate in a study about making healthy changes. Participants were recruited from the local community in South-Western Ontario, Canada, with flyers, newspaper ads, and a recruitment booth at the local mall. Potential participants were first screened for eligibility, which was that they were planning to make one or more health behaviour changes in the next 6 months, and they had not already started to make these changes. Those who met the inclusion criteria were given a survey package (by mail or in person depending on the point of initial contact) to complete and return by mail along with a signed consent form. Participants were recruited at the mall through a small table set up with signs. Due to constraints from the mall management, participants were not actively approached and contact was made only if potential participants approached the recruitment table. Participants who returned the completed survey package were compensated for their time with a $15 mall gift card.

Fourteen participants who didn’t comply with the instructions for generating HPS were removed, leaving a final sample of 191 (67.5% female; Mean age = 34.03, SD = 13.9). Participants were predominantly white, the majority had a university education; however, there was a range of income levels across participants. Full demographic characteristics of the sample are presented in Table 1 The data analysed for the current study is from Time 1 of a larger two time-point prospective study (Sirois and Giguère 2018).

**Table 1:** Socio-demographic characteristics of the sample

*N*	191
Sex (% female)	67.5
Age	
Mean (SD)	34.03 (13.9)
Range	18–73
Ethnicity (% Caucasian)	80.6
Income (Canadian $)	
Less than 20,000	18.9
21,000 to 40,000	17.9
41,000 to 60,000	13.2
61,000 to 80,000	13.2
81,000 to 100,000	7.9
Greater than 100,000	13.9
Prefer not to answer	14.7
Employment status (%)	
Full-time	38.3
Part-time	26.4
Unemployed/retired	30.1
Disabled	5.2
Education (%)	
High school or less	19.3
University or college	67.1
Graduate school	13.0
Relationship status (%)	
Married/living with partner	53.1
Separated/Divorced/Widowed	12.0
Never married	34.9
Diagnosed with a psychiatric condition (% yes)	21.6

*SD* Standard deviation

### Measures

In addition to demographic questions, participants completed a set of measures. Only those analysed for the current research are reported.

#### Screening Question about Health Behaviours

Potential participants were asked “Are you intending to make healthy changes within the next 6 months?” and given examples of heathy changes (i.e, eating healthier, exercising more regularly, reducing stress). Those who answered “yes” were then asked if they had started to work on these changes. Those who answered “yes” were excluded, and those who answered “no” were invited to participate in the study.

#### Health-Related Possible Selves

Participants were instructed to generate three hoped-for-HPS and feared-HPS in free text boxes using instructions adapted from Hooker (1992), and list them order of importance. They then rated their efficacy (“How capable do you feel of achieving/avoiding this possible self in the future?”), and outcome (“How likely is it that this possible self will be achieved/avoided in the future?”), expectancies for their most important hoped-for-HPS and feared-HPS on two items with a 5-point scale (*not at all capable/likely* to *definitely capable/likely*) adapted from Hooker (1992). The items were identical to those used by Hooker (1992), apart from the original rating scale which was 7-point. This adjustment was made so that the interim anchors on the scale could be more easily understandable, and to out the scale on a similar metric to the procrastination scale.

#### Trait Procrastination

The 20-item Lay’s General Procrastination Scale (GPS; Lay 1986) assessed global tendencies towards chronic procrastination across a variety of tasks (e.g., In preparing for some deadlines, I often waste time by doing other things.) Agreement with each item is rated on a 5-point Likert scale (*Strongly disagree* to *Strongly agree*), with items averaged into a single score such that high values indicate a greater tendency to procrastinate. The GPS has demonstrated excellent test-retest reliability over a 10 year period (Steel 2007), and good internal consistency (alpha = 0.82; Lay 1986). Internal consistency for the current study was good, α = 0.88.

#### Motivation and Intentions to Make Health Behaviour Changes

Participants were instructed to list up to three health behaviour changes they wanted to make over the next 6 months, and rank one as most important. For their most important health behaviour change, they rated the strength of their intentions (“How strong are your intentions to actually follow through and start to change this behaviour within the next 6 months?”) and motivations (“How motivated do you feel to try and change this health behaviour?”) on two items, each rated on a 9-point Likert scale ranging from 1 (*no intention/not at all motivated*) to 9 (*very strong intentions/extremely motivated*). These single item measures were chosen as it was important to assess motivation and intentions specific to the intended health behaviour changes rather than assessing general intentions and motivations. This choice was consistent with previous research that has demonstrated that constructs that are more concrete and less complex or abstract can be reliably assessed with single item adjective rating scales (Bergkvist and Rossiter 2007; Zimmerman et al. 2006), and that such single-item scales perform as well as their longer multi-item counterparts (Bergkvist and Rossiter 2007).

### Data Analysis

All data were first checked for normality. Apart from the health behaviour intentions variable, which showed some signs of skewness in the histogram, all variables were normally distributed. Missing data was handled via listwise deletion. Correlation analysis was used to test the proposed bivariate relations among the model variables. Parallel mediation models were planned to test the significance of the indirect effects of trait procrastination on the two dependent variables health behaviour change 1) intentions and 2) motivations through efficacy and outcome expectancies. These two models were tested for both hoped-for-HPS and feared-HPS using the SPSS macro PROCESS (Hayes, 2013). Analyses were conducted with both mediators entered simultaneously with 10,000 bootstrapping re-samples and bias corrected 95% confidence intervals (bcCI). Age and participant sex were entered as covariates in the models to control for their effects on both mediators and the dependent variables.

## Results

The health behaviour changes people listed as being most important focused predominantly on diet and exercise related changes. Participants generated a variety of different hoped-for-HPS and feared-HPS which broadly focused on key health behaviours such eating healthy, being physically active, and managing stress better, as well as avoiding disease and disability due to poor health (see Table 2 for examples).

**Table 2:** Examples of hoped for and feared health–related possible selves generated by the study participants

Hoped-for possible selves	Feared possible selves
Being a vegetarian	Being someone with cancer
Able to handle stress more efficiently	Being obese
Physically active and healthy eater	Having a heart attack
At least 25 lbs. lighter (hopefully more!)	Being in a wheelchair
Living out a long & healthy life	Gaining weight back
Bring someone with a healthy life-style	Becoming very ill
Being physically stronger than I am now	Unhealthy, weak woman

Correlation analysis using Pearson’s *r* revealed the expected negative associations of trait procrastination with efficacy and outcome expectancies for hoped-for-HPS, but not feared-HPS (Table 3). Trait procrastination was also significantly associated with weaker intentions and motivations to make the most important health behaviour change. Hoped-for-HPS expectancies were positively associated with health behaviour change intentions and motivations. Similar to previous research (Beutel et al. 2016), trait procrastination was negatively associated with age, *r* = .20, *p* = .007.

**Table 3:** Descriptive statistics and pearson correlations among trait procrastination, Hoped-for and Feared Health-Related Possible Selves (HPS) Expectancies, and Health Behaviour Change Intentions and Motivations

Variable	1	2	3	4	5	6	7
1. Trait procrastination	–						
2. Efficacy expectancies – hoped-for HPS	−.153*	–					
3. Outcome expectancies – hoped-for HPS	−.198**	.603**	–				
4. Efficacy expectancies – feared HPS	.052	.160*	.177*	–			
5. Outcome expectancies – feared HPS	−.071	.230**	.322**	.662**	–		
6. Intentions to change health behaviour	−.218**	.201**	.319**	−.043	.086	–	
7. Motivations to change health behaviour	−.222**	.234**	.249**	−.043	.094	.647**	
Mean	2.46	3.65	3.57	3.23	3.26	7.20	7.24
Standard deviation	0.64	0.88	0.85	0.98	0.93	1.20	0.96

*p < .05; **p < .01

**Fig. 2:**
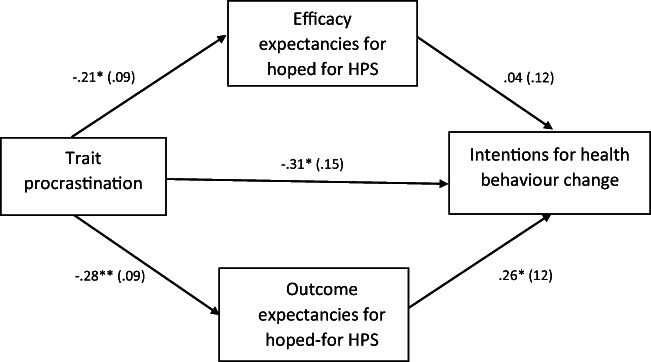
Results for the model of the indirect effects of trait procrastination on intentions for making health behaviour changes through efficacy and outcome expectancies for achieving hoped-for health-related possible selves (HPS). Path coefficients are unstandardized. Participant sex and age were included as covariates. *p < .05; ** *p* < .01

Given the lack of significant associations of feared-HPS with procrastination, motivations and intentions, tests of the indirect effects model (Fig. 2) were only conducted for hoped-for-HPS efficacy and outcome expectancies. The test of the indirect effects of trait procrastination on health behaviour intentions was significant for hoped-for-HPS outcome expectancies, but not efficacy expectancies (see Table 4). The direct effect of trait procrastination was no longer significant after accounting for the two expectancy mediators. The overall model explained 7% of the variance in health behaviour intentions. For health behaviour motivations, the indirect effects for both efficacy and outcome expectancies were not significant.

**Table 4:** Indirect effects of Trait Procrastination (TP) via Efficacy Expectancies (EE) and Outcome Expectancies (OE) on Health Behaviour Change Intentions (HBI) and Motivations (HBM)

Path	B (*SE*)	*t*	BCA CIs	Model *R*^2^	*F* (*df*)
Panel A: Health behaviour intentions					
TP – EE (a_1_)	−.21 (.09)	−2.14*			
Age – EE	−.00 (.00)	−0.39			
Sex – EE	.16 (.13)	1.16			
TP – OE (a_2_)	−.28 (.09)	−3.13**			
Age – OE	−.00 (.00)	−0.35			
Sex – OE	.14 (.13)	1.00			
EE – HBI (b_1_)	.04 (.12)	0.33			
OE – HBI (b_2_)	.26 (.12)	2.23*			
Age – HBI	.00 (.01)	0.12			
Sex – HBI	−.11 (.22)	−0.51			
Total effect: TP – HBI (c)	−.39 (.14)	−2.91**		.07	3.92** (3, 187)
Age – HBI	.00 (.01)	0.07			
Sex – HBI	−.07 (.22)	−0.34			
Direct effect: TP – HBI (c’)	−.31 (.15)	−2.04			
Indirect effect: TP – EE– HBI	−.01 (.03)		[−.08, .03]		
Indirect effect: TP – OE– HBI	−.08 (.05)		[−.20, −.01]		
Panel B: Health behaviour motivations					
TP – EE (a_1_)	−.21 (.10)	−2.11*			
Age – EE	−.00 (.00)	−0.28			
Sex – EE	.15 (.13)	1.15			
TP – OE (a_2_)	−.28 (.09)	−3.13**			
Age – OE	−.00 (.00)	−0.21			
Sex – OE	.13 (.14)	0.95			
EE – HBM (b_1_)	.23 (.15)	1.53			
OE – HBM (b_2_)	.05 (.18)	0.30			
Age – HBM	.01 (.01)	1.82			
Sex – HBM	−.14 (.25)	−0.56			
Total effect: TP – HBM (c)	−.37 (.17)	−2.17*		.05	3.70* (3, 187)
Age – HBM	.01 (.01)	1.79			
Sex – HBM	−.07 (.22)	−0.34			
Direct effect: TP – HBM (c’)	−.31 (.19)	−1.65			
Indirect effect: TP – EE– HBM	−.05 (.04)		[−.17, .00]		
Indirect effect: TP – OE– HBM	−.02 (.05)		[−.12, .09]		

BCA CI = Bias corrected and accelerated 95% confidence intervals; Boot strapping analysis was conducted with 10,000 resamples; all effects are unstandardized; age and sex were included as covariates. *p < .05, **p < .01

## Discussion

This aim of the current study was to examine the contributions of efficacy and outcome expectancies for HPS in explaining the associations of trait procrastination to intentions and motivations for making intended health behaviour changes. The findings were somewhat consistent with possible selves theory (Markus and Nurius 1986) and with self-efficacy theory (Bandura 1977) in that trait procrastination, a chronic tendency towards self-regulation difficulties, was negatively associated with outcome and efficacy expectancies for achieving hoped-for-HPS, which reflect self-regulatory processes proposed to be necessary to achieve the health goals captured by HPS. However, low expectancies, and not low efficacy expectancies, that HPS would be achieved explained the association between procrastination and weak intentions to make intended health behaviour changes, after controlling for potential age and gender differences. The findings did not support the proposition that trait procrastination would be linked to lower outcome and efficacy expectancies for avoiding a feared-HPS, or that such expectancies would be associated with health behaviour intentions and motivations.

The asymmetrical association of procrastination with expectancies for the hoped-for-HPS and feared-HPS and in turn health behaviour intentions and motivations, parallels Hooker’s (1992) findings that expectancies for avoiding feared-HPS explained little variance in perceived health in comparison to those for hoped-for HPS. In this respect the current findings provide further evidence that the self-regulation processes involved in pursuing approach (hoped-for-HPS) and avoidance (feared-HPS) health goals may operate differently. It is likely that the behaviours needed to achieve hoped-for-HPS may be clearer than those for avoiding feared-HPS. For example, “Becoming a vegetarian” implies eliminating certain foods from one’s diet, whereas becoming “someone with cancer” has less obvious links to the behaviours needed to avoid having this feared-HPS become a reality. This may explain why, despite trait procrastination being related to avoidant coping strategies (Sirois and Kitner 2015), trait procrastination was not significantly associated with expectancies for avoiding feared-HPS. This explanation is also consistent with research demonstrating that avoidance health goals are more difficult to achieve than approach health goals, and thus may require behaviour change tools such as implementation intentions (Sullivan and Rothman 2008). The non-significant correlation between procrastination and expectancies for avoiding feared-HPS may simply reflect the fact that feared-HPS do not have clear links to the specific behaviours needed to avoid these HPS, making it difficult to estimate the associated efficacy and outcome expectancies. Because the scores on procrastination and the outcome and efficacy expectations were normally distributed, it is unlikely that the null findings were due to floor or ceiling effects in the values.

The current findings are consistent with previous research noting that trait procrastination is associated with difficulty in prospective and future-oriented thinking (Liu and Feng 2018; Sirois 2014), and is linked to low self-efficacy within the health domain (Sirois 2004). The current research builds on this by demonstrating that procrastinators also have problems feeling capable of taking action to realize the health goals embodied by their hoped-for-HPS and feared-HPS, and perceive that realizing such goals may be less likely. This in turn may limit the potential of these possible selves to set behavioural standards for guiding self-regulation aimed at improving health (e.g., vanDellen and Hoyle 2008), and thus contribute to the difficulties that chronic procrastinators have in successfully achieving their health behaviours (Sirois 2007).

That the indirect effects for outcome expectancies but not efficacy expectancies were significant is intriguing and in contrast to other research which generally finds that efficacy expectations are the stronger predictor of health behaviours (e.g., Anderson et al. 2006). However, it should be noted that in the current study the outcome and efficacy expectancies were with respect to achieving a hoped-for-HPS, and not engaging in a specific health behaviour. In Bandura’s (1977) model of self-efficacy, efficacy expectancies play a role in linking the person to the behaviour, such that feeling one is capable of engaging in the behaviour will promote engaging in the behaviour. In contrast, outcome expectancies reflect the likelihood that engaging in a specific behaviour will lead to the desired outcome. In the possible selves paradigm, both efficacy and outcome expectancies refer to achieving or avoiding a HPS, rather than a specific behaviour and its related outcomes. If we consider that HPS can be conceived of as embodying a variety of health behaviours necessary to make the hoped-for-HPS a reality (e.g., eating healthier, exercising regularly), and as an outcome to be achieved (e.g., being a healthier person), then it seems reasonable that the pattern of results for these expectancies might differ from previous work.

The current findings have implications for understanding the self-regulatory process associated with the cognitive representations of health goals made by people with chronic self-regulation issues, and how they might be improved to strengthen health behaviour change intentions. When such individuals do not believe that a possible healthy self can be achieved, intentions to follow through even with health behaviours that are self-chosen may be low. Using a mental imagery intervention focused on increasing the vividness and empathetic engagement with a future possible self is one approach shown to be effective for reducing procrastination in general (Blouin-Hudon and Pychyl 2017). This approach may also be useful for strengthening expectations that a future, hoped-for HPS can actually be achieved, and as suggested by the current findings, strengthen subsequent health behaviour intentions.

Although this study provides a first test of how trait procrastination, an individual difference reflecting self-regulation difficulties, relates to HPS, the current findings should be considered within the context of several limitations. Analyses were based on cross-sectional data, limiting inferences about the directionality of the relations among the model variables. Participants were also highly educated, and pre-existing health conditions were not screened for, although roughly one fifth of participants reported being diagnosed with a psychiatric condition. However, that the results were found despite not excluding participants with health conditions that may have affected their HPS, speaks to the robustness and generalisability of the findings across diverse health conditions. In addition, trait procrastination as measured by the GPS (Lay 1986) is considered a relatively stable trait with a moderate degree of heritability (46%) demonstrated in behaviour-genetics research with over 300 same-sex twin pairs (Gustavson et al. 2014). This, and theory on possible selves (Markus and Nurius 1986) and their prospective relations with health behaviours (Murru and Martin Ginis 2010), supports the temporal order suggested by the model tested. Nonetheless, longitudinal research that considers not just intentions and motivations, but also actual behaviours, would provide more compelling support for the relationships suggested by the current findings. In addition, the effects sizes of the paths were relatively small as was the indirect effect for health behaviour intentions. One notable strength of the current study was that participants were intending to engage in health behaviour changes over the next 6 months. This likely increased the salience and relevance of their HPS for their intentions and motivations for health behaviour change, which referred to self-chosen behaviours, and thus increased the ecological validity of the findings.

In conclusion, the current study extends the limited evidence regarding the role of expectancies for HPS in health behaviour change, and provides new insights into how personality traits linked to self-regulation difficulties are associated with HPS self-regulatory processes. Trait procrastination was associated with lower outcome and efficacy expectancies for realising hoped-for-HPS, and lower HPS outcome expectancies explained weaker intentions for engaging in a self-chosen health behaviour change. The results from this study and from previous work suggest that interventions that promote vividly envisioning a hoped-for-HPS to increase expectations that such HPS can be achieved may help bolster intentions to engage in health behaviour change, and thus be a fruitful avenue for future research.
